# U-shaped relationship between birth weight and childhood blood pressure in China

**DOI:** 10.1186/s12887-019-1638-9

**Published:** 2019-07-31

**Authors:** Chong Lai, Yiyan Hu, Di He, Li Liang, Feng Xiong, Geli Liu, Chunxiu Gong, Feihong Luo, Shaoke Chen, Chunlin Wang, Yimin Zhu

**Affiliations:** 1 Department of Surgery, The First Affiliated Hospital, Zhejiang University School of Medicine, Hangzhou, China; 2 Department of Epidemiology & Biostatistics, Zhejiang University School of Public Health, Hangzhou, China; 3 Department of Pediatrics, The First Affiliated Hospital, Zhejiang University School of Medicine, Hangzhou, China; 4 Department of Endocrinology, Chongqing Medical University Affiliated Children’s Hospital, Chongqing, China; 5 Department of Pediatrics, Tianjin Medical University General Hospital, Tianjin, China; 6 Department of Pediatrics, Beijing Children’s Hospital Affiliated to Capital Medical University, Beijing, China; 7 Department of Pediatric Endocrinology and Genetic Metabolic Diseases, Children’s Hospital of Fudan University, Shanghai, China; 8 Department of Pediatrics Endocrinology, Maternal and Child Health Hospital of Guangxi Zhuang Autonomous Region, Nanning, China

**Keywords:** Birth weight, Obesity, Childhood blood pressure, Systolic blood pressure, Diastolic blood pressure, Hypertension

## Abstract

**Background:**

The relationship between birth weight and blood pressure has not been well explored in Chinese children and adolescents. The aim of this study was to investigate the relationship between birth weight and childhood blood pressure in China.

**Methods:**

A total of 15324 children and adolescents (7919 boys and 7405 girls) aged 7–17 years were stratified into six birth weight groups. Analysis of covariance and binary logistic regression were used to analyse the relationship between birth weight and blood pressure while controlling for potential confounding factors, including age, gestational age, season of birth and area of residence.

**Results:**

The group with birth weights from 2500 to 2999 g had the lowest prevalence of hypertension (8.9%). Lower birth weight children (< 2000 g) had significantly higher systolic blood pressure (SBP) (106.00 ± 0.72, *P* = 0.017), and children with heavier birth weights also had higher SBP (3500–3999 g, 105.13 ± 0.17, *P <* .001; ≥ 4000 g, 105.96 ± 0.27, *P <* .001). No significant relationship was found between birth weight and diastolic blood pressure (DBP). The overall rate of hypertension was 10.8% (12.1% in boys and 9.4% in girls). The median weight group (2500–2999 g) had the lowest rate of hypertension (8.9%). Compared with children in the median weight group, children with lower birth weight had a higher prevalence of hypertension (< 2000 g, OR = 1.85, 95% CI = 1.25–2.74; 2000–2499 g, OR = 1.57, 95% CI = 1.15–2.13), and groups with higher birth weights also had higher risks of hypertension (3500–3999 g, OR = 1.22, 95% CI = 1.02–1.45; ≥ 4000 g, OR = 1.42, 95% CI = 1.16–1.74).

**Conclusions:**

Excluding the confounding effect of obesity, a U-shaped relationship between birth weight and risk of hypertension was found in children and adolescents in Chinese cities. Birth weight significantly influences SBP but has a minimal effect on DBP. Further basic research on foetal development and programming may shed light on this phenomenon.

## Background

There is a consensus that cardiovascular function and blood pressure are determined during childhood and continue into adulthood [1]. Childhood hypertension has been considered a strong predicative factor for hypertension in adulthood. In the Beijing blood pressure cohort study, by measuring the inter-vessel parameters, Liang et al. followed 1259 subjects (6–18 years old) over 24 years and found that children with elevated blood pressure had accelerated remodelling of both cardiac and arterial systems in early and middle adulthood [2]. Targeted organ damage, especially damage to the heart, was detectable in some hypertensive children [3]. Therefore, childhood hypertension should now be considered a public health concern in younger generations both in developed countries and in developing countries with rapid development.

Childhood blood pressure is affected both by genetic and environmental factors, including factors at birth (birth weight) and factors after birth (dietary structure, weight, physical activity) [4–6]. Poor weight management and childhood metabolic syndrome have been demonstrated as some of the leading causes of abnormal blood pressure in children [7]. China is experiencing a huge increase in the prevalence of obesity among children and adolescents. Cao et al. first observed that the incidence of hypertension was 3.1% among teenagers (12–17 years old) in Changsha, and the risk of hypertension increased three- to four-fold once BMI reached above the 95th percentile [8]. Dong et al. explored the relationship between BMI and blood pressure in Chinese children. After statistical adjustment for BMI, the mean increase in SBP was reduced by 40.5%, which indicated that obesity was one of the leading determinants of high SBP [9]. Moreover, the residual increase suggested that some other important factors also contributed to childhood hypertension.

The developmental origins of health and disease (DOHaD) theory and the life course theory (LCT) lead to the hypothesis that nutritional status in utero may permanently change the body’s structure, function and metabolism in ways that cause chronic diseases in later life [10–12]. There is some evidence showing that people born with high birth weight are at higher risk of developing cardiovascular diseases in later life [13]. Xie et al. collected cross-sectional data from 1253 female nurses aged 35–65 years and found a J-shaped relationship between birth weight and blood pressure in adulthood [14]. Launer et al. found that both low birth weight and high birth weight infants had a higher risk of elevated blood pressure [15]. However, the results from Chinese studies were quite inconsistent with the previous conclusions. When Zhai et al. analysed 18920 students aged 6–11 years, they found that elevated blood pressure in children and teenagers was associated only with BMI but not with birth weight [16]. Li et al. selected 1435 pairs of children with high or normal birth weight from a birth cohort between 1993 and 1995 in Wuxi and followed them until 2005 to 2007. They found no statistically significant relationship between high birth weight and blood pressure [17].

In the present study, we analysed data from a metabolic syndrome investigation among children and adolescents in six cities across China and adjusted for the influence of BMI and other confounders. We finally demonstrated the potential influence of birth weight on childhood blood pressure. The purpose of the current study was first to investigate the prevalence of hypertension in children and adolescents in China. Second and more importantly, we aimed to reveal the association between birth weight and childhood hypertension.

## Methods

### Subjects

Subjects were recruited from a school-based cluster investigation of metabolic syndrome among children and adolescents in six provincial capitals in China in September 2010 [18]. The initial aim of this cross-sectional study was to investigate the incidence and prevalence of metabolic syndrome and obesity among children and adolescents. A total of 17035 participants, aged 7–17 years, were recruited for this study. A total of 121 subjects with cancer, chronic diseases (heart, lung, and kidney) or severe acute infections were excluded. Of the participants, 1590 lacked information about birth weight for personal reasons. Therefore, 15324 subjects with complete information on birth weight and blood pressure were analysed. The protocol of this study was proved by the Research Ethics Committee of the School of Public Health and the Medical Ethics Committees at the Children’s Hospital and the First Affiliated Hospital of the Zhejiang University College of Medicine.

### Data collection and measurements

Well-trained investigators measured anthropometric indices, including weight and height, following a standard protocol, referring to a previous study [18]. Information on demographic variables, including sex, date of birth, gestational age, area of residence and parental information, was collected through face-to-face interviews with the simultaneous presence of the participants and their parents. Blood pressure was measured three times in a sitting position with a cuff haemadynamometer after sitting quietly for 5 min. Parents were asked to provide the official birth certificates of their children for the record of birth weight and gestational age.

### Definitions and potential confounding factors

Body mass index (BMI) was calculated as body weight in kilograms divided by height in metres squared (kg/m^2^). The BMI reference material issued by the World Health Organization (WHO) in 2007 (for individuals 5–19 years old) was adopted to establish the BMI Z-scores (BAZ) [19]. Systolic blood pressure (SBP) was defined by the first Korotkoff sound and diastolic blood pressure (DBP) by the fourth Korotkoff sound. The values of SBP and DBP were calculated by the average of three repeated measurements. Gestational age was determined as the number of completed weeks of gestation from the last menstrual period (LMP) to the date of birth. If there was a significant difference between gestational age estimated by LMP and the ultrasound results, the ultrasound estimate was used. We used the final data on the birth records of the subjects, which were recorded by obstetricians. Hypertension was defined as being above the 95th percentile of each category based on different ages and sexes, according to the cut-offs of the Beijing standards for Chinese children and adolescents (3–17 years old) [20]. Either outlier SBP or outlier DBP was defined as hypertension. Subjects were divided into six categories by birth weight in grams with 500 g intervals: < 2000 g, 2000–2499 g, 2500–2999 g, 3000–3499 g, 3500–3999 g, and ≥ 4000 g.

### Statistical analyses

Normally distributed variables are expressed as the mean ± standard deviation (SD) and were compared by Student’s t test. Categorical variables are expressed by frequencies (percentages) and were compared by χ^2^ tests. The analysis of covariance was used to correct the covariate effects and to compare differences in blood pressure among birth weight groups. The group with birth weights of 2500–2999 g was chosen as a reference because this group of children had the lowest blood pressures and lowest prevalence of hypertension. A Dunn-Bonferroni test was applied for post hoc comparisons. Binary logistic regression analysis was used to explore the influence of birth weight on high blood pressure or hypertension. Age, gestational age, BAZ, season of birth and area of residence were regarded as confounding factors, which were adjusted for in the comparisons (as footnoted under the tables). Because BMI or BAZ have long been recognized as core and volatile factors influencing blood pressure, a two-step adjustment was conducted. Confounding factors excluding BAZ were first adjusted (estimated marginal mean1 ± SE1), and BAZ was subsequently adjusted for along with the other factors (estimated marginal mean2 ± SE2). The quadratic and cubic models were used as simulators of curve estimation. All tests were two-sided, and the results were considered significant when the *p*-value was ≤0.05. Statistical analyses were performed using SPSS for Windows (SPSS 17.0 Inc., Chicago, IL).

## Results

### Basic characteristics of the subjects

The demographic data and anthropometric variables of the subjects were stratified by sex and are listed in Table 1. The information of 15324 subjects aged 7–17 years was analysed, and among the subjects, 7919 were boys and 7405 were girls. Subjects were recruited from six advanced Chinese cities, Chongqing (20.2%), Hangzhou (20.8%), Nanning (16.9%), Beijing (11.9%), Shanghai (10.1%) and Tianjin (20.1%). The sex distribution at each age was not significantly different. There was no notable difference between boys and girls in gestational age or season of birth. Boys had higher birth weight, weight and height (*P* < .001). The prevalence of hypertension in boys was significantly higher than in girls (12.1% vs. 9.2%, *P <* .001).

**Table 1:** Demographic data and anthropometric variables of the subjects^a^

Variables	Total	Boys	Girls	P^b^
	*N* = 15,324	*N* = 7919	*N* = 7405	
Age (y), n (%)	11.54 ± 2.59	11.49 ± 2.60	11.59 ± 2.58	.579
7	938 (6.1)	506 (6.4)	432 (5.8)	.220
8	1444 (9.4)	763 (9.6)	681 (9.2)	
9	1571 (10.3)	818 (10.3)	753 (10.2)	
10	1843 (12.0)	957 (12.1)	886 (12.0)	
11	1618 (10.6)	861 (10.9)	757 (10.2)	
12	1734 (11.3)	908 (11.5)	826 (11.2)	
13	1998 (13.0)	979 (12.4)	1019 (13.8)	
14	2059 (13.4)	1042 (13.2)	1017 (13.7)	
15	1358 (8.9)	695 (8.8)	663 (9.0)	
16	576 (3.8)	288 (3.6)	288 (3.9)	
17	185 (1.2)	102 (1.3)	83 (1.1)	
Weight (kg)	41.42 ± 14.40	42.54 ± 15.63	40.21 ± 12.84	< 0.001
Height (cm)	147.07 ± 15.49	147.89 ± 16.73	146.20 ± 13.99	< 0.001
BMI (kg/m^2^)	18.58 ± 3.68	18.82 ± 3.89	18.33 ± 3.43	< 0.001
BAZ	< 0.001 ± 1.00	0.06 ± 1.05	−0.07 ± 0.93	< 0.001
Birthweight (g)	3281.0 ± 522.9	3338.9 ± 534.8	3219.2 ± 502.7	< 0.001
Gestational age (wk)	38.44 ± 4.70	38.44 ± 4.81	38.43 ± 4.59	.391
Systolic blood pressure (mmHg)	104.61 ± 12.10	106.08 ± 12.46	103.04 ± 11.51	< 0.001
Diastolic blood pressure (mmHg)	65.12 ± 8.25	65.56 ± 8.62	64.64 ± 8.01	< 0.001
Hypertension, n (%)				< 0.001
Normal	13666 (89.2)	6958 (87.9)	6708 (90.6)	
Abnormal	1658 (10.8)	961 (12.1)	697 (9.4)	
Birth weight category (g), n (%)				< 0.001
< 2000	236 (1.5)	124 (1.6)	112 (1.5)	
2000–2499	490 (3.2)	217 (2.7)	273 (2.7)	
2500–2999	2398 (15.6)	1066 (13.5)	1332 (18.0)	
3000–3499	6445 (42.1)	3084 (38.9)	3361 (45.4)	
3500–3999	4095 (26.7)	2350 (29.7)	1745 (23.6)	
≥ 4000	1660 (10.8)	1078 (13.6)	582 (7.9)	
Area of residence, n (%)				.890
Chongqing	3094 (20.2)	1550 (19.6)	1544 (20.8)	
Hangzhou	3185 (20.8)	1842 (23.6)	1343 (18.1)	
Nanning	2592 (16.9)	1328 (16.8)	1264 (17.1)	
Beijing	1832 (11.9)	928 (11.7)	904 (12.2)	
Shanghai	1545 (10.1)	771 (9.7)	774 (10.4)	
Tianjin	3076 (20.1)	1500 (18.9)	1576 (21.3)	
Season of Birth, n (%)				.322
Spring	3563 (23.3)	1866 (23.6)	1697 (22.9)	
Summer	3834 (25.0)	1976 (25.0	1858 (25.1)	
Autumn	4050 (26.4)	2097 (26.5)	1953 (26.4)	
Winter	3877 (25.3)	1980 (25.0)	1897 (25.6)	

Season of birth: spring = infants born in March, April and May; summer = infants born in June, July and August; autumn = infants born in September, October and November; winter = infants born in December, January and February

*SD* Standard deviation

^a^Quantitative data are expressed as the mean ± SD (standard deviation), and qualitative data are expressed as frequency (%)

^b^P for t tests or χ^2^ tests

### Birth weight and systolic blood pressure

**Fig. 1:**
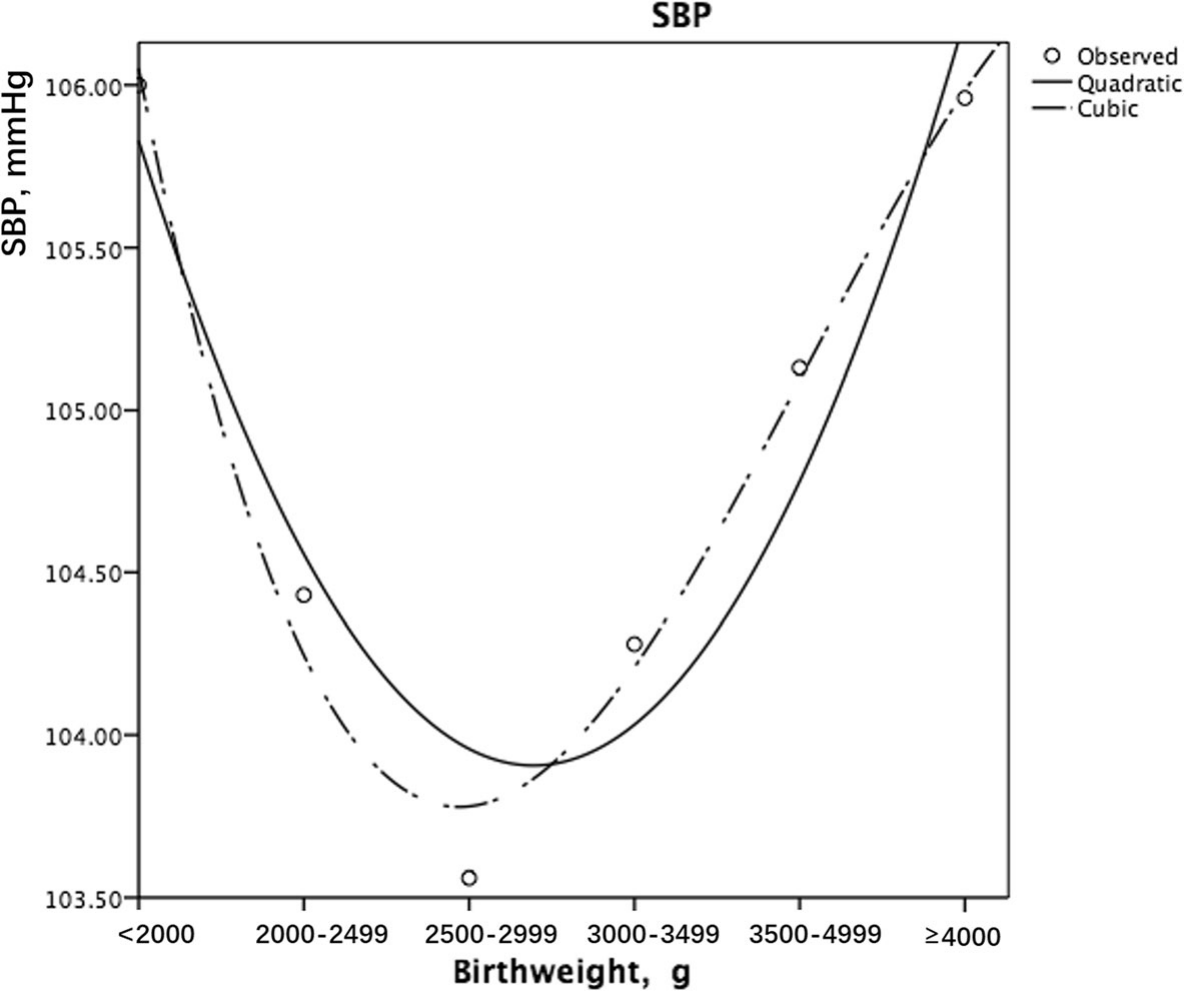
Curve estimation of the association between birth weight and SBP (the quadratic and the cubic modelling both showed a U-shaped association)

Table 2 presents the associations between birth weight and blood pressure. After adjustment for confounders, for the whole population, the median birth weight group (2500–2999 g) had the lowest blood pressure (SBP: 103.56 ± 0.23; DBP: 64.55 ± 0.16), and therefore, we set this group as the reference. Low birth weight subjects (< 2000 g) had a significantly higher SBP (106.00 ± 0.72, *P* = .017), while children with birth weights over 3500 g also had higher SBP (3500–3999 g, 105.13 ± 0.17, *P* < .001; ≥ 4000 g, 105.96 ± 0.27, *P* < .001). The additional adjustment for BAZ did not change the association between birth weight and blood pressure. The quadratic or cubic model estimated a U-shaped association between birth weight and SBP, even after the adjustment for BAZ (Fig. 1). When stratified by sex, we found a J-shaped association between birth weight and SBP for each gender group. Boys with birth weights over 4000 g had higher SBP (107.18 ± 0.34, *P <* .001). Girls with birth weights over 3500 g also had higher SBP (3500–3999 g, 103.97 ± 0.26, *P <* .001; ≥ 4000 g, 104.72 ± 0.45, *P <* .001). Boys or girls with extremely low birth weight did not show significant SBP differences when compared with the reference group. However, the adjusted mean was higher than that of the normal group, suggesting that the statistical insignificance might be due to the small sample size.

**Table 2:** The association between birth weight and blood pressure based on the analysis of covariance

Birth weight, g		N	Systolic blood pressure (SBP)								Diastolic blood pressure (DBP)							
			Mean	SD	Estimated marginal means 1^a^	SE1	P1^c^	Estimated marginal means 2^b^	SE2	P2^d^	Mean	SD	Estimated marginal means 1^a^	SE1	P1^c^	Estimated marginal means 2^b^	SE2	P2^d^
Total	< 2000	236	104.01	12.26	105.54	0.74	.038	106.00	0.72	.017	65.12	8.27	65.82	0.53	.120	66.08	0.52	.075
	2000–2499	490	103.88	12.04	103.90	0.51	1.000	104.43	0.50	1.000	64.64	8.47	64.65	0.37	1.000	64.95	0.36	1.000
	2500–2999	2398	102.99	11.87	103.19	0.23	Ref	103.56	0.23	Ref	64.28	8.28	64.34	0.17	Ref	64.55	0.16	Ref
	3000–3499	6445	104.15	11.86	104.19	0.14	.004	104.28	0.14	.092	64.87	8.30	64.87	0.10	.092	64.93	0.10	.755
	3500–3999	4095	105.37	12.32	105.37	0.18	< 0.001	105.13	0.17	< 0.001	65.52	8.33	65.55	0.13	< 0.001	65.41	0.13	0.001
	≥ 4000	1660	107.16	12.33	106.49	0.28	< 0.001	105.96	0.27	< 0.001	66.40	8.44	66.16	0.20	< 0.001	65.86	0.20	< 0.001
Boys	< 2000	124	105.18	12.28	106.97	1.04	.598	107.72	1.00	.219	65.40	9.02	66.20	0.76	1.000	66.61	0.74	1.000
	2000–2499	217	105.28	12.54	105.25	0.79	1.000	105.88	0.76	1.000	64.62	8.47	64.64	0.57	1.000	64.99	0.56	1.000
	2500–2999	1066	104.60	12.22	104.70	0.36	Ref	105.14	0.34	Ref	64.99	8.75	65.03	0.26	Ref	65.27	0.25	Ref
	3000–3499	3084	105.78	12.36	105.70	0.21	.226	105.83	0.20	1.000	65.41	8.63	65.38	0.15	1.000	65.44	0.15	1.000
	3500–3999	2350	106.39	12.61	106.52	0.24	< 0.001	106.27	0.23	.087	65.69	8.54	65.73	0.17	.350	65.60	0.17	1.000
	≥ 4000	1078	107.98	12.44	107.64	0.35	< 0.001	107.18	0.34	< 0.001	66.50	8.55	66.38	0.26	.003	66.12	0.25	.253
Girls	< 2000	112	102.72	12.16	103.92	1.04	.573	104.12	1.02	.638	64.80	7.39	65.40	0.74	.415	65.53	0.73	.462
	2000–2499	273	102.78	11.53	102.52	0.67	1.000	102.96	0.65	1.000	64.66	8.48	64.56	0.48	1.000	64.83	0.47	.972
	2500–2999	1332	101.70	11.42	101.67	0.30	Ref	101.97	0.29	Ref	63.71	7.85	63.70	0.22	Ref	63.89	0.21	Ref
	3000–3499	3361	102.65	11.18	102.60	0.19	.140	102.66	0.19	.719	64.38	7.95	64.35	0.14	.152	64.39	0.13	.639
	3500–3999	1745	104.00	11.79	104.20	0.26	< 0.001	103.97	0.26	< 0.001	65.29	8.03	65.39	0.19	< 0.001	65.25	0.18	< 0.001
	≥ 4000	582	105.64	11.98	105.32	0.46	< 0.001	104.72	0.45	< 0.001	66.21	8.23	66.05	0.33	< 0.001	65.68	0.32	< 0.001

*SD* Standard deviation, *SE* Standard error, *Ref* Reference

^a^Calculated in the analysis of covariance after adjusting for age, gestational age, area of residence, and season of birth

^b^Additional adjustment for BAZ

^c^based on estimated marginal means 1; reference: birth weight 2500–2999 g

^d^based on estimated marginal means 2; reference: birth weight 2500–2999 g

### Birth weight and diastolic blood pressure

**Fig. 2:**
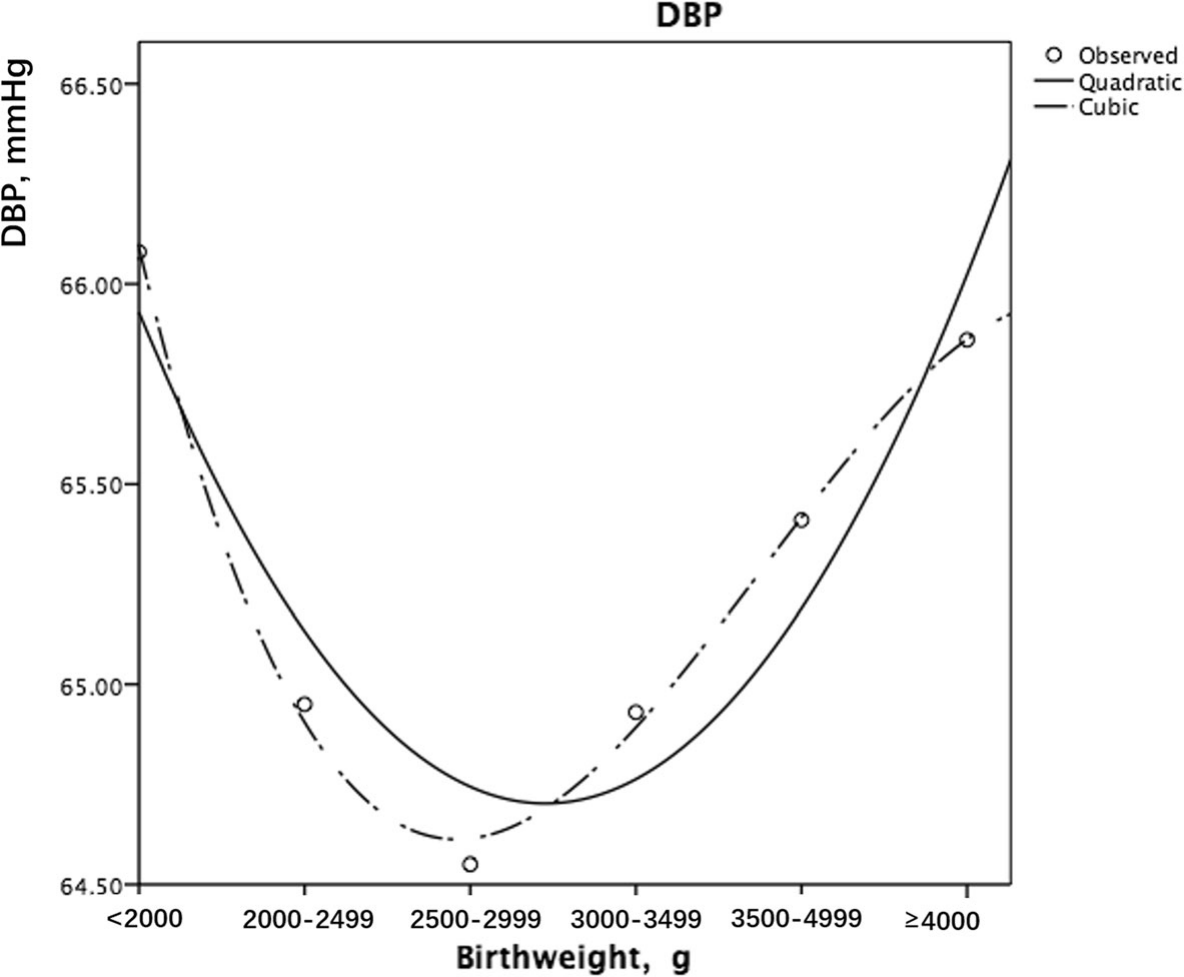
Curve estimation of the association between birth weight and DBP (the quadratic and the cubic modelling both showed a U-shaped association)

The association between birth weight and DBP was also U-shaped among the different birth weight groups when BAZ was controlled (Fig. 2). The low birth weight group (< 2000 g) had a higher DBP (66.08 ± 0.52, *P* = .075), while those with a birth weight over 3500 g also had a higher DBP (3500–3999 g, 65.41 ± 0.13, *P =* .001; ≥ 4000 g, 65.86 ± 0.20, *P <* .001). When stratified by sex, the association became nonsignificant. Girls with birth weights over 3500 g had higher DBP (3500–3999 g, 65.25 ± 0.18, *P <* .001; ≥ 4000 g, 65.68 ± 0.32, *P <* .001).

### Birth weight and hypertension

**Fig. 3:**
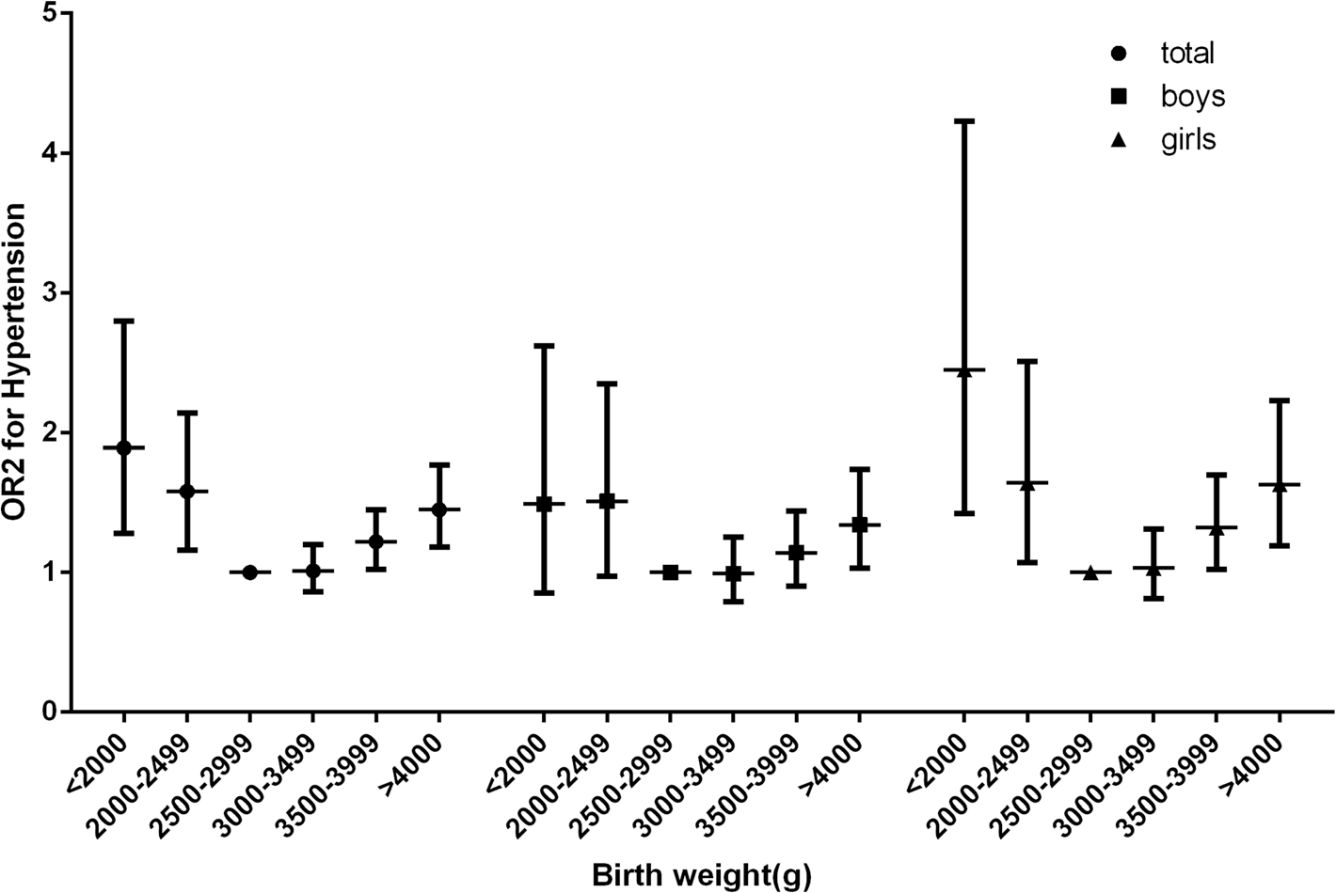
Odds ratios of different birth weight groups for hypertension

**Fig. 4:**
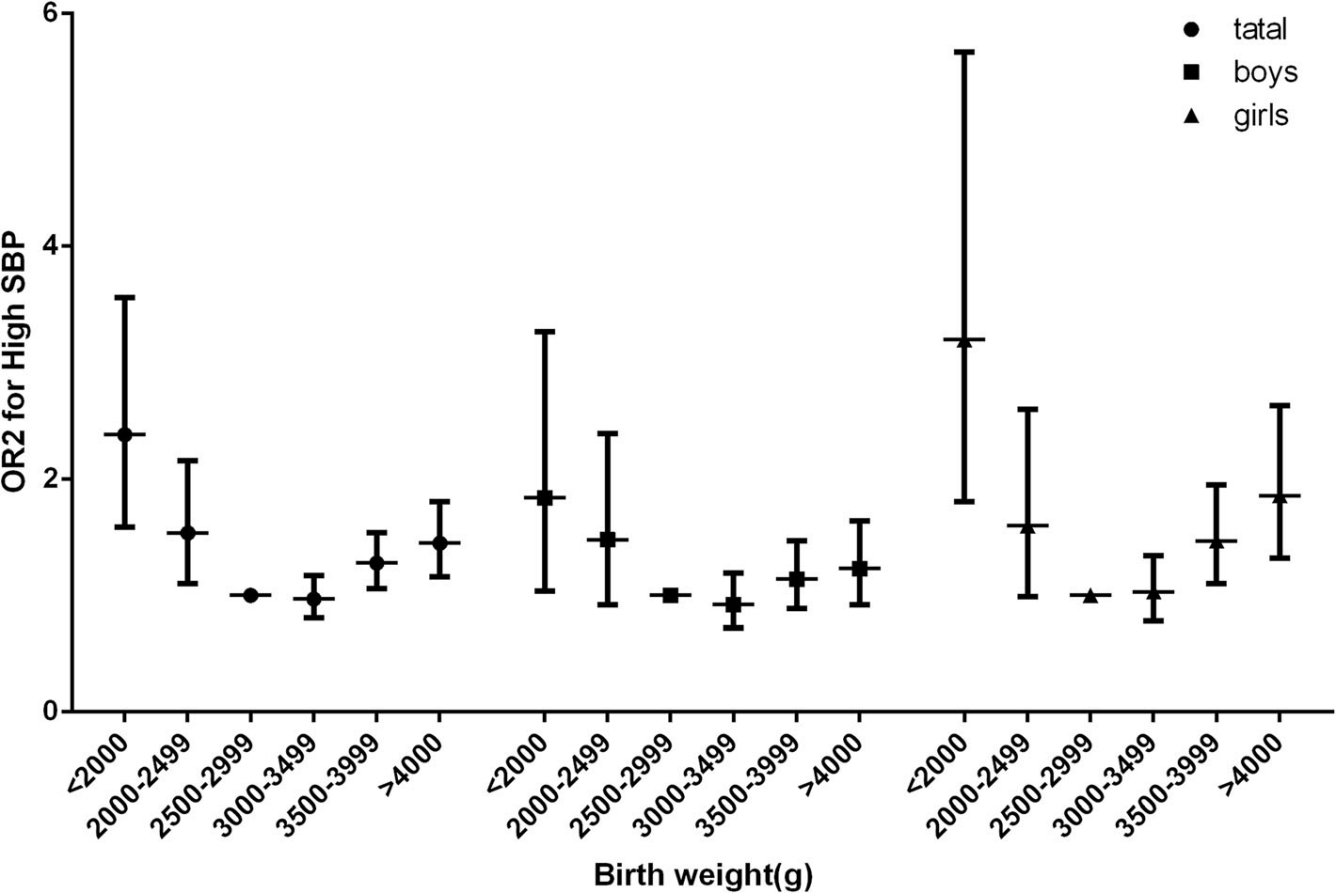
Odds ratios of different birth weight groups for high SBP

The prevalence of hypertension in different birth weight groups is shown in Table 3. The overall rate of hypertension was 10.8% (12.1% in boys and 9.4% in girls) in the target population. A U-shaped association was found between birth weight and the prevalence of hypertension (Fig. 3). The median birth weight group (2500–2999 g) had the lowest prevalence of hypertension (8.9%). Subjects with birth weights lower than 2500 g had a higher prevalence of hypertension (< 2000 g, OR = 1.85, 95% CI = 1.25–2.74; 2000–2499 g, OR = 1.57, 95% CI = 1.15–2.13). Subjects with birth weights higher than 3500 g also had a higher risk of hypertension (3500–3999 g, OR = 1.22, 95% CI = 1.02–1.45; ≥ 4000 g, OR = 1.45, 95% CI = 1.16–1.74). When separated by sex, the results were consistent with the trend of the whole population. High SBP and high DBP were subsequently analysed. The low birth weight group (≤ 2500 g) had a higher prevalence of high SBP (< 2000 g, OR = 2.33, 95% CI = 1.53–3.50; 2000–2499 g, OR = 1.53, 95% CI = 1.08–2.15), and subjects with birth weights greater than 3500 g also had higher risks of high SPB (3500–3999 g, OR = 1.28, 95% CI = 1.06–1.55; ≥ 4000 g, OR = 1.42, 95% CI = 1.14–1.77) (Fig. 4). However, no significant differences were found among birth weight groups when considering the prevalence of high DBP after performing the adjustments. Neither the low birth weight group nor the high birth weight group showed any disparities.

**Table 3:** The association between birth weight and hypertension based on binary logistic regression

Birth weight, g		High SBP, n (%)		OR1 (95% CI)^a^	P1^c^	OR2 (95% CI)^b^	P2^d^	High DBP, n (%)		OR1 (95% CI)^a^	P1^c^	OR2 (95% CI)^b^	P2^d^	Hypertension, n (%)		OR1 (95% CI)^a^	P1^c^	OR2 (95% CI)^b^	P2^d^
		Normal	Abnormal					Normal	Abnormal					Normal	Abnormal				
Total	< 2000	201 (85.2)	35 (14.8)	2.17 (1.46–3.23)	< 0.001	2.33 (1.53–3.50)	< 0.001	223 (94.5)	13 (5.5)	1.07 (0.58–1.96)	.832	1.10 (0.60–2.03)	.750	200 (84.7)	36 (15.3)	1.75 (1.19–2.58)	.005	1.85 (1.25–2.74)	.002
	2000–2499	440 (89.8)	50 (10.2)	1.47 (1.05–2.05)	.024	1.53 (1.08–2.15)	.015	460 (93.9)	30 (6.1)	1.46 (0.96–2.23)	.077	1.46 (0.96–2.24)	.079	427 (87.1)	63 (12.9)	1.51 (1.12–2.04)	.007	1.57 (1.15–2.13)	.004
	2500–2999	2226 (92.8)	172 (7.2)	Ref	–	Ref	–	2294 (95.7)	104 (4.3)	Ref	–	Ref	–	2184 (91.1)	214 (8.9)	Ref	–	Ref	–
	3000–3499	5965 (92.6)	480 (7.4)	1.04 (0.87–1.25)	.675	0.98 (0.82–1.18)	.835	6114 (95.3)	301 (4.7)	1.08 (0.86–1.36)	.499	1.02 (0.81–1.29)	.864	5831 (90.5)	614 (9.5)	1.08 (0.91–1.27)	.375	1.02 (0.86–1.20)	.823
	3500–3999	3679 (89.8)	416 (10.2)	1.42 (1.18–1.71)	< 0.001	1.28 (1.06–1.55)	.010	3866 (94.4)	229 (5.6)	1.25 (0.98–1.59)	.067	1.13 (0.89–1.44)	.323	3606 (88.1)	489 (11.9)	1.34 (1.13–1.59)	.001	1.22 (1.02–1.45)	.025
	≥4000	1460 (88.0)	200 (14.8)	1.65 (1.33–2.05)	< 0.001	1.42 (1.14–1.77)	.002	1564 (94.2)	96 (5.8)	1.31 (0.98–1.75)	.064	1.12 (0.84–1.50)	.429	1418 (85.4)	242 (14.6)	1.64 (1.35–2.00)	< 0.001	1.42 (1.16–1.74)	.001
Boys	< 2000	107 (86.3)	17 (13.7)	1.69 (0.96–2.96)	.098	1.87 (1.05–3.33)	.033	118 (95.2)	6 (4.8)	0.77 (0.32–1.86)	.567	0.83 (0.34–2.01)	.682	107 (86.3)	17 (13.7)	1.39 (0.80–2.41)	.251	1.51 (0.86–2.66)	.155
	2000–2499	191 (88.0)	26 (12.0)	1.42 (0.89–2.26)	.136	1.47 (0.91–2.38)	.117	203 (93.5)	14 (6.5)	1.23 (0.67–2.25)	.511	1.24 (0.67–2.30)	.497	186 (85.7)	31 (14.3)	1.45 (0.94–2.23)	.093	1.51 (0.96–2.35)	.072
	2500–2999	972 (91.2)	94 (8.8)	Ref	–	Ref	–	1009 (94.7)	57 (5.3)	Ref	–	Ref	–	955 (89.6)	111 (10.4)	Ref	–	Ref	–
	3000–3499	2814 (91.2)	270 (8.8)	1.01 (0.79–1.29)	.956	0.93 (0.73–1.20)	.596	2923 (94.8)	161 (5.2)	0.98 (0.72–1.34)	.914	0.92 (0.67–1.26)	.608	2747 (89.1)	337 (10.9)	1.07 (0.85–1.34)	.550	1.00 (0.80–1.27)	.973
	3500–3999	2091 (89.0)	259 (11.0)	1.29 (1.01–1.65)	.045	1.15 (0.89–1.48)	.288	2207 (93.9)	143 (6.1)	1.14 (0.83–1.57)	.405	1.02 (0.74–1.41)	.903	2048 (87.1)	302 (12.9)	1.28 (1.01–1.61)	.040	1.14 (0.90–1.45)	.266
	≥4000	947 (87.8)	131 (12.2)	1.40 (1.06–1.85)	.019	1.21 (0.91–1.62)	.185	1016 (94.2)	62 (5.8)	1.10 (0.76–1.60)	.618	0.95 (0.65–1.38)	.781	915 (84.9)	163 (15.1)	1.53 (1.18–1.98)	.001	1.32 (1.02–1.72)	.038
Girls	< 2000	94 (83.9)	18 (16.1)	3.06 (1.73–5.41)	< 0.001	3.14 (1.76–5.60)	< 0.001	105 (93.8)	7 (6.2)	1.50 (0.65–3.48)	.359	1.51 (0.65–3.51)	.338	93 (83.0)	19 (17.0)	2.33 (1.34–4.02)	.003	2.37 (1.36–4.12)	.002
	2000–2499	249 (91.2)	24 (8.8)	1.56 (0.96–2.53)	.069	1.62 (0.99–2.64)	.054	257 (94.1)	16 (5.9)	1.74 (0.96–3.13)	.066	1.73 (0.96–3.13)	.069	241 (88.3)	32 (11.7)	1.62 (1.05–2.45)	.029	1.66 (1.08–2.54)	.022
	2500–2999	1254 (94.1)	78 (5.9)	Ref	–	Ref	–	1285 (96.5)	47 (3.5)	Ref	–	Ref	–	1229 (92.3)	103 (7.7)	Ref	–	Ref	–
	3000–3499	3151 (93.8)	210 (6.2)	1.08 (0.82–1.41)	.594	1.03 (0.79–1.35)	.830	3221 (95.8)	140 (4.2)	1.19 (0.85–1.67)	.311	1.13 (0.81–1.60)	.473	3084 (91.8)	277 (8.2)	1.08 (0.85–1.36)	.541	1.03 (0.81–1.31)	.794
	3500–3999	1588 (91.0)	157 (9.0)	1.59 (1.20–2.11)	.001	1.46 (1.10–1.95)	.009	1659 (95.1)	86 (4.9)	1.38 (0.96–1.98)	.087	1.26 (0.87–1.82)	.215	1558 (89.3)	187 (10.7)	1.43 (1.11–1.84)	.006	1.32 (1.02–1.70)	.035
	≥4000	513 (88.1)	69 (11.9)	2.11 (1.50–2.97)	< 0.001	1.81 (1.28–2.57)	.001	548 (94.2)	34 (5.8)	1.42 (0.89–2.25)	.023	1.42 (0.90–2.26)	.135	503 (86.4)	79 (13.6)	1.84 (1.35–2.51)	< 0.001	1.58 (1.15–2.18)	.004

*SBP* Systolic blood pressure, *DBP* Diastolic blood pressure, *SD* Standard deviation, *SE* Standard error, *OR* Odds ratio, *CI* Confidence interval, *Ref* Reference

^a^Calculated in the binary logistic regression analysis after adjusting for age, gestational age, area of residence, season of birth

^b^Additional adjustment for BAZ.

^c^based on OR1; reference: birth weight at 2500–2999 g

^d^based on OR2; reference: birth weight at 2500–2999 g

## Discussion

The current study examined the association between birth weight and childhood blood pressure by collecting school-based data from six Chinese cities. This study is also the first, to the best of our knowledge, to investigate the relationship between birth weight and childhood hypertension using a large census from urban areas in China. In summary, birth weight had a profound impact on childhood blood pressure and the prevalence of primary hypertension in Chinese children and adolescents. Moreover, the association between birth weight and blood pressure remained U-shaped after adjusting for various confounding factors, including BAZ, season of birth and area of residence. Children with birth weights from 2500 to 2999 g had the lowest blood pressure and lowest risk for childhood hypertension. Birth weight significantly influenced systolic blood pressure. However, its effect on diastolic blood pressure remains unknown.

Some of the previous studies suggested an inverse relationship between birth weight and childhood blood pressure, while others showed a positive relationship or no association at all. In 2012, Edvardsson et al. reviewed the existing studies and listed several reasons that might drive the results apart. They believed that inadequate adjustment for potential confounders, failure to use standard blood pressure values, and disparities in the target populations together contributed to the discrepant results [21]. The results from the current study verified a U-shaped relationship between birth weight and childhood blood pressure. The associations between birth weight and childhood blood pressure were not unidirectional, and this, to some extent, explained why neither the inverse nor the positive modelling was adequate to explain the true relationship in reality.

The proposed mechanisms linking birth weight and childhood hypertension have been widely studied. As has been shown in animal models and partly in humans, the hyperfiltration theory suggests that the reduction in nephron number, a decreased kidney mass and a reduction in renal reserve in low birth weight children enhance salt sensitivity and increase the risk of hypertension [22–24]. The mechanism linking high birth weight to childhood hypertension was buried within the correlation between birth weight and current weight. Metabolic syndrome and obesity play important roles in the development of arterial stiffness and endothelial dysfunction [7, 25, 26]. However, as presented in this study, current weight or BAZ is not adequate to explain the increase in blood pressure. The Barker theory posits that cardiovascular diseases originate during intra-uterine development and that undernutrition in utero permanently changes the organ structure, function and systematic metabolism in ways that lead to cardiovascular events in later life [10]. Pietrobelli et al. further expanded the Barker hypothesis and suggested that intra-uterine nutritional status should be intervened upon artificially to avoid childhood obesity and coronary artery diseases in the future [27]. Hence, foetal programming needs to be studied more extensively to determine the underlying pathophysiological mechanisms. Of interest was that DBP was not influenced by birth weight, emphasizing the possibility of different mechanisms behind high SBP and high DBP in children and adolescents. Traditionally, DBP is considered the most important component of blood pressure. However, there are no studies on isolated DBP levels in either adults or children. In studies of the ageing population, SBP and pulse pressure (SBP - DBP) have been considered to be better predictors of cardiac risks [28]. The results here showed that BMI and birth weight influenced DBP but failed to explain its elevation above the normal range.

As even small increases in blood pressure are known to increase the long-term risk of cardiovascular diseases and hypertensive nephropathy, it is crucial to understand the aetiology of primary childhood hypertension and to look for potential precautions. Li et al. reported that the prevalence of abnormal blood pressure, together with obesity, dramatically increased from 1993 to 2013 in China [29]. Paediatric hypertension is usually asymptomatic, difficult to recognize by parents and can easily be missed by health professionals. Moreover, even pre-hypertension is not completely benign, and its rate of progression to hypertension is approximately 7% per year over a 2-year interval [3]. The relationship between birth weight and hypertension increases from childhood to adulthood [30]. According to the conclusions of this study, the prevention of primary hypertension may require more insight into foetal development and birth weight control in a reasonable range. In the era of precise medicine, it is promising to intervene in the risk factors during the gestational stage or early childhood. Prevention of cardiovascular diseases should begin in childhood by regularly screening for hypertension, counselling for healthy lifestyle habits and avoiding preventable risk factors.

In this work, the study population was well defined, and we used Chinese-specific standardized methods to collect data. The effects of main potential confounders, especially BAZ, were controlled in the analysis of covariance. However, the present study had limitations. This was a cross-sectional study, and there might be some recall bias in the interview results. Second, information on growth patterns was not collected. An increasing amount of evidence is available showing that birth weight can influence childhood growth velocity and influence blood pressure [31, 32]. Third, a lack of information on physical activity and the socioeconomic status of each family was collected. Researchers have found socioeconomic status to be an important risk factor both for birth weight and blood pressure [33, 34]. For the reasons mentioned above, well-designed prospective studies are urgently needed to examine infants and track their blood pressure into adulthood to verify the causal effect of birth weight on hypertension. Information about family history, physical exercise, pubertal development and socioeconomic status should be clearly recorded and taken into analysis.

## Conclusions

This study revealed that birth weight was associated with blood pressure levels and the risk of hypertension in Chinese children and adolescents. Both low and high birth weight increased the risk of hypertension. Birth weight influenced SBP but had a minimal effect on DBP.

## Data Availability

All data generated or analysed during this study are included in this published article: Zhou D, Yang M, Yuan Z, Zhang D, Liang L, Wang C, et al. Waist-to-Height Ratio: a simple, effective and practical screening tool for childhood obesity and metabolic syndrome. Prev Med. 2014;37:35–40.

## Abbreviations

BAZ: BMI Z-scores
BMI: Body mass index
CI: Confidence interval
DBP: Diastolic blood pressure
DOHaD: The developmental origins of health and disease theory
LCT: Life course theory
OR: Odds ratio
Ref: Reference
SBP: Systolic blood pressure
SD: Standard deviation
SE: Standard error
WHO: World Health Organization
